# Marine Pollutant Tributyltin Affects DNA Methylation and Fitness of Banded Murex (*Hexaplex trunculus*) Populations

**DOI:** 10.3390/toxics11030276

**Published:** 2023-03-17

**Authors:** Maja Šrut, Iva Sabolić, Anita Erdelez, Dorotea Grbin, Martina Furdek Turk, Robert Bakarić, Melita Peharda, Anamaria Štambuk

**Affiliations:** 1 Institute of Zoology, Center for Molecular Biosciences, University of Innsbruck, Technikerstraße 25, 6020 Innsbruck, Austria; maja.srut@uibk.ac.at; 2 Department of Biology, Faculty of Science, University of Zagreb, Rooseveltov trg 6, 10000 Zagreb, Croatia; iva.sabolic@gmail.com (I.S.); dorotea.polo@gmail.com (D.G.); rbakaric17@gmail.com (R.B.); 3 Institute of Oceanography and Fisheries, Šetalište I. Meštrovića 63, 21000 Split, Croatia; aerdelez@gmail.com (A.E.); melita@izor.hr (M.P.); 4 Division for Marine and Environmental Research, Ruđer Bošković Institute, Bijenička 54, 10000 Zagreb, Croatia; martina.furdek@irb.hr

**Keywords:** population epigenetics, fitness, imposex, pollution, evolution

## Abstract

Banded murex, *Hexaplex trunculus*, is a marine gastropod whose reproductive fitness can be severely affected by very low concentrations of antifouling compound tributyltin (TBT). TBT has strong xenoandrogen impacts on snails, causing the development of imposex (e.g., the superimposition of male sexual characteristic in females), thereby affecting the fitness of entire populations. TBT is also known as a DNA-demethylating agent and an obesogenic factor. The aim of this study was to unravel the interactions between TBT bioaccumulation, phenotypic responses, and epigenetic and genetic endpoints in native populations of *H. trunculus*. Seven populations inhabiting environments along the pollution gradient were sampled in the coastal eastern Adriatic. These included sites of intense marine traffic and boat maintenance activity and sites with low anthropogenic impact. Populations inhabiting intermediately and highly polluted sites exhibited higher TBT burdens, higher incidences of imposex, and higher wet masses of snails than populations in lowly polluted sites. Other morphometric traits and cellular biomarker responses did not show clear differentiation among populations in relation to marine traffic/pollution intensity. An analysis of methylation sensitive amplification polymorphism (MSAP) revealed environmentally driven population differentiation and higher epigenetics than genetic within-population diversity. Moreover, decreases in genome-wide DNA methylation coincided with the imposex level and snail mass, suggesting an epigenetic background of the animal phenotypic response.

## 1. Introduction

Anthropogenic disturbances are progressively changing environmental conditions in all ecosystems. In response to altered environments, organisms can adapt, acclimatise, migrate, or die out. Emerging evidence suggests that in addition to genetic variation, epigenetic variation plays an important role in species’ ability to cope with environmental change [1]. Epigenetic modifications can be readily induced in a within-generation timeframe, and they are often linked to immediate phenotypic responses [2]. They also ensure an additional level of variation for selection to act upon, and they are to some extent heritable [3,4,5]. However, the understanding of environment–epigenetic interactions is hindered by the responsive manner in which epigenetic marks can be directed by environmental cues, resulting in phenotypes that are not only better fitted to the environment but also stochastically altered by it, causing non-adaptive phenotypic alterations and decreased fitness in general. As evidence accumulates on the importance of epigenetic–environment interactions, our interest in understanding the patterns of epigenetic diversity in natural populations, its adaptive or maladaptive significance, and the epigenetic consequences of anthropogenic change in particular is steadily increasing.

One of the most striking examples of anthropogenically induced compounds affecting the fitness of natural populations is the case of tributyltin (TBT). Since their discovery in 1960s, organotin compounds (OTCs) (particularly TBT) have been widely used as the main ingredients of antifouling paints for ships and fishing nets. However, it quickly became evident that TBT is a strong endocrine disruptor whose effects extend to untargeted species, causing severe outcomes for marine wildlife [6]. One of the most profound effects of TBT on marine gastropods is the induction of imposex, an irreversible superimposition of male sexual characters on females. This phenomenon occurs even at environmental concentrations as low as several nanograms of TBT per litre [7,8]. Low imposex stages are recognised by the onset of penis growth in females, and at more advanced stages, fully grown penises and vas deferentia are observed. Final imposex stages result in vas deferens closing the vaginal opening and/or splitting the capsule gland. This can substantially affect female reproductive abilities and might lead to severe recruitment failure in affected populations. In some gastropod species, such as *Nucella lapillus*, TBT-induced imposex is known to have caused a complete population collapse [9]. Despite the total ban on TBT use in European countries by the International Convention on the Control of Harmful Anti-fouling Systems on Ships (AFS Convention) since 2008, recent data suggest that TBT still persists in the environment due to the resuspension of contaminated sediment and recurrent recent input [10,11,12,13,14,15]. Although several temporal studies have documented a decrease in imposex occurrence linked to the TBT ban, ongoing TBT environmental input is corroborated by many recent studies still reporting high incidences of imposex in studied gastropod populations [12,16,17]. TBT is degraded in marine environments to less toxic dibutyltin (DBT) and monobutyltin (MBT), but its exact half-life greatly varies according to environmental conditions [18]. TBT is also known as DNA-hypomethylating agent, causing non-adaptive epigenetic alterations. TBT exposure has been shown to decrease genome-wide and gene-specific DNA methylation in various animals, including mammals, fish and molluscs. This effect has been coupled with altered gene expression and/or adipocyte differentiation [19,20,21,22], and in some cases genome-region-specific hypo- or hyper methylation has been observed [23]. Due to interference with adipocyte differentiation, TBT has also been termed model obesogen [24].

Environmental epigenetics using marine invertebrates as suitable sentinel organisms represents an innovative and ground-breaking approach for improving the management and conservation of natural marine resources [25]. Banded murex (*Hexaplex trunculus*) is a common Mediterranean gastropod. Due to its benthic lifestyle, limited mobility, crawling larvae (lacking the planktonic larval stage), and ability to develop imposex at TBT concentrations as low as 1 ng Sn g^−1^, it has been described as the most reliable indicator of TBT pollution [26,27]. The impact of TBT on the reproductive fitness of *H. trunculus* has been extensively studied for decades. However, the link between environmental exposure to TBT, DNA methylation changes, and organism phenotypic responses has not been examined to date. Therefore, the aim of this study was to unravel the relationships among TBT burden, epigenetic and genetic patterns, and phenotypic responses in natural populations of *H. trunculus.*

In this study, the effects of TBT on genome-wide DNA methylation were examined with the aim to provide important insights into the DNA methylation effects in native murex populations upon environmental TBT exposure and their link to reproductive fitness. This study also questioned if and how environmental TBT exposure affects epigenetic population differentiation and diversity. The environment usually impacts the overall genetic makeup of affected populations over the course of generations through the forces of natural selection (i.e., genotypic change is driven by phenotypes defining individual reproductive or adaptive success), altered mutation rates, genetic drift, and altered gene flow. However, in terms of epigenetic responses, one would expect immediate, within-generation local effects. In addition to examining population reproductive fitness, further phenotypic characteristics that might show responses toward TBT load (biomarkers, mass, and morphometrics) were also studied to broaden the comprehension of TBT’s overall biological impact on the phenotypes in natural populations.

## 2. Material and Methods

### 2.1. Study Area

**Figure 1:**
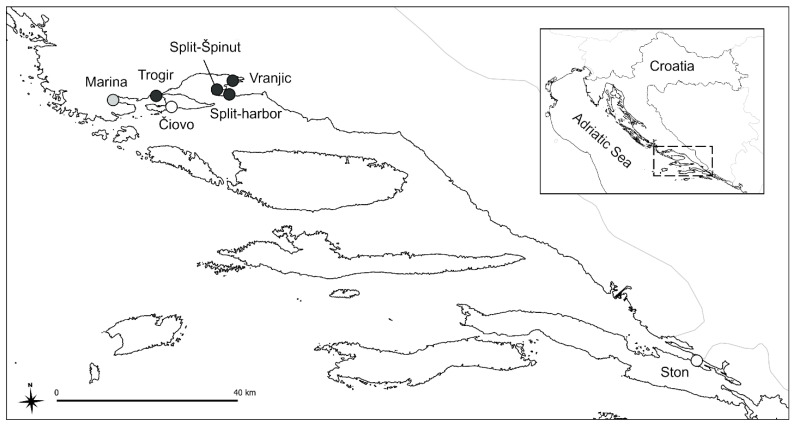
Map indicating sampling sites. Lowly polluted sites (in white): Čiovo and Ston; intermediately polluted site (in grey): Marina; highly polluted sites (in black): Trogir, Vranjic, Split-Špinut, and Split-harbour.

Marine gastropod banded dye-murex (*Hexaplex trunculus*) specimens were collected in April and May 2014 at seven locations along the pollution gradient in the mid and south Adriatic (Figure 1). 

**Figure 2:**
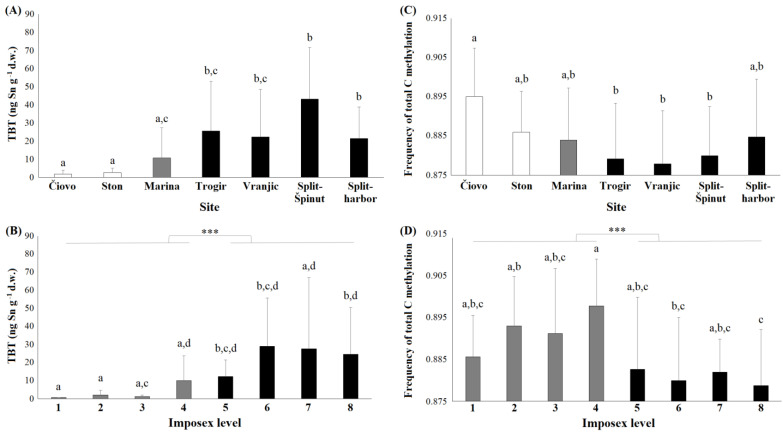
TBT concentration and DNA methylation across populations and imposex levels of banded murex (*H. trunculus*) collected in the Adriatic Sea. TBT concentrations (ng Sn g^−1^ dry weight) measured in whole *H. trunculus* tissue: (**A**) TBT per sampling site; (**B**) TBT per imposex level, mean ± SD. Letters relate to the significance between populations/groups (Mann–Whitney U test, *p* < 0.05), and stars (***) denote the significance between low (1–4) and high (5–8) imposex levels (Mann–Whitney U test, *p* < 0.001). Frequency of DNA methylation: (**C**) per sampling site; (**D**) per females exhibiting specific imposex level (from normal females denoted as level 1 to females with the highest imposex denoted as level 8). Letters relate to the significance between populations/groups (ANOVA and Tukey’s post hoc test, *p* < 0.05), and stars (***) denote the significance between low (1–4) and high (5–8) imposex levels (ANOVA and Tukey’s post hoc test, *p* < 0.001). (**A**,**C**) White bars indicate sites of low pollution pressure, grey bars indicate sites of intermediate pollution pressure, and black bars indicate sites of high pollution pressure (**B**,**D**) Grey bars indicate low (1–4) and black bars indicate high (5–8) imposex levels.

The sites with low levels of marine traffic activities, Čiovo and Ston, are referred to throughout the manuscript as sites of low pollution intensity, and Marina, with moderate marine activity (mostly small private boats), is referred to as an intermediately polluted site. Sites within ports and marinas with intense marine traffic activities—Trogir, Vranjic, Split-Špinut, and Split-harbour—are referred to as highly polluted sites. This classification was confirmed by TBT concentrations in the sediment. Sediment samples collected in 2015 revealed a TBT concentration of around 40 ng Sn g^−1^ at the Marina site, whereas the level recorded at the Ston site was below the detection limit [27]. The amount recorded at the Marina site corresponded to a moderately polluted location based on the previously proposed categorization of sites based on TBT sediment concentrations: 4–18, 22–73, and 109–365 ng Sn g^−1^ represent low, moderate, and high TBT pollution levels, respectively [28]. Low, intermediate, and high TBT-related pollution levels of sites was further confirmed by the accumulation of TBT in snail tissue (see Figure 2A). 

At each site, between 48 and 88 (Table S1) adult murex snails (shell length > 40 mm) were collected by scuba diving at depths of 1–10 m. Upon sampling, murex snails were transferred to the Laboratory of Fisheries Science and Management of Pelagic and Demersal Resources at the Institute of Oceanography and Fisheries in Split. Murex snails were kept in marine water for maximum of 48 h until further processing. 

### 2.2. Determination of Organotin Compounds 

The measured organotin compounds (OTCs) included TBT and its degradation derivative dibutyltin (DBT). OTCs in whole murex tissue were determined in 8–10 females per population following the previously described analytical procedure [10]. Briefly, butyltins were extracted from 1 g of lyophilised tissue with 0.1 mol/L HCl in methanol using ultrasound-assisted stirring (30 min and 55 Hz). The simultaneous derivatisation and extraction step was performed in a sodium acetate–acetic acid buffer (pH = 4.8) with NaBEt_4_ (1%, *w*/*v*) and hexane via mechanical shaking at 350 rpm for 30 min. Analyses were carried out on a gas chromatograph (GC, Varian CP 3800) with a pulsed flame photometric detector (PFPD, Varian). Quality control was performed by analysing the certified reference materials (CRM) for mussels (CE 477, ERM, European Commission, Geel, Belgium). Measurements were performed in two technical replicates, and the mean value of two replicates was used for further analyses. Data are presented as mean ± SD across populations. The Mann–Whitney U test was used to perform statistical comparisons due to non-normally distributed data (level of significance: *p* ≤ 0.05).

### 2.3. Phenotypic Responses

#### 2.3.1. IMPOSEX Determination 

Imposex was determined in 215 females (24–36 individuals per population), following the previously described recommendations [26,29], as described in Table 1. This 8-stage imposex progression scale, in which vas deferens bypasses the vaginal opening (stage 4.3) and proliferates along the capsule gland (4.7) instead of directly overgrowing the vulva and vagina and causing sterility, is typical for *H. trunculus* individuals [26]. Each imposex stage was transferred to the corresponding imposex level (1–8), with the aim to have an ordinal response variable for the purpose of running an ordered logit regression model. Data are presented as the percentage of each imposex level in each population.

In order to test whether the imposex level (ordinal response variable) could be predicted by either TBT tissue accumulation or DNA methylation (predictors), an ordered logit regression model was applied using the “polr” function available in the “MASS” R package (R studio version 1.0.136). The model was considered significant at *p* ≤ 0.05. The use of ordered logit regression was proposed to model ordinal variables in environmental monitoring studies [30].

#### 2.3.2. Biomarker Activity Measurements

Biomarker measurements (catalase, glutathione reductase, glutathione S-transferase, acetylcholinesterase, carbonyl, and lipid peroxidation) were conducted on the digestive gland tissue of 6–10 individuals per population in accordance with well-established protocols for marine invertebrates, with slight modifications [31,32,33,34,35,36,37]. The detailed protocols are described in the Supporting Information. Values for each biomarker were normalised based on the protein content in each sample. Principal component analysis (PCA) was performed using the “prcomp” function (“stats” R package) in R studio 1.0.136. An ANOVA and Tukey’s post hoc test were applied to the PC1 and PC2 scores in order to define significance in population separation using the “aov” and “TukeyHSD” functions, respectively, from the “stats” R package (*p* ≤ 0.05).

#### 2.3.3. Morphometric Measurements

For each individual, several morphometric measures were recorded using a digital calliper with a precision of 0.1 mm: shell length (TL), shell breadth (TB), shell opening breadth (AW), and shell opening length (AL). Total mass (TM) was recorded using a digital balance with a precision of 0.1 g. Upon the careful removal of the murex shell, the total mass of the soft tissue (WM) and the mass of the gonads and hepatopancreas (GHM) were recorded. Murex snails were dissected in order to determine their sex and imposex level in females by making a longitudinal cut along the hypobranchial gland. Males were distinguished by a penis located behind the right ocular tentacle, the presence of vas deferens, and (more specifically) the absence of a capsular gland and vulva, which are typical female characteristics [26,38]. In order to avoid measuring bias, morphometric analyses were performed on murex snails from all populations except for Čiovo, as these individuals were processed by a different researcher. 

All morphometric measurements were log-transformed and normalised to the total length. PCA was separately performed on male and female individuals in R studio 1.0.136. An ANOVA and Tukey’s post hoc test were applied to the PC1 and PC2 scores in order to define significance in population separation using the “aov” and “TukeyHSD” functions, respectively, from the “stats” R package (*p* ≤ 0.05). The means of each morphometric measure per population for males and females were separately transformed in a population pairwise proximity matrix with Ward’s method based on squared Euclidean distances using cluster analysis in SPSS Statistics 23.0 (IBM). Proximity matrices were correlated with a matrix of geographic distances between populations and matrices of population pairwise FST values for genetic and epigenetic data using the Mantel test. The test was performed using the “mantel.rtest” function in the “ade4” R package.

Data on the total soft tissue mass (WM) are presented as mean ± SD. Statistical comparisons were performed using an ANOVA (data are normally distributed) followed by Tukey’s post hoc test using the “aov” and “TukeyHSD” functions, respectively, in R studio (*p* ≤ 0.05). In order to test whether the WM (response variable) could be predicted by TBT tissue accumulation or DNA methylation (predictors), a linear regression model was applied. The effect of imposex level (predictor) on WM (response) was tested using a one-way ANOVA. We employed the same method to test whether individual- or population-level TBT burdens were good predictors of genome-wide DNA methylation. Both the linear regression model and the one-way ANOVA were applied using the “lm” R function. The model was considered significant at *p* ≤ 0.05.

### 2.4. Methylation Sensitive Amplification Polymorphism (MSAP)

MSAP was performed on 30 individuals per population. Genomic DNA from 150 mg of murex foot tissue was extracted using the GenElute Mammalian DNA miniprep kit (Sigma-Aldrich, Germany) according to the manufacturer’s instructions. The standard protocol for MSAP was followed [39] with slight modifications. Restriction digestion and an adapter ligation of 250 ng genomic DNA were simultaneously performed at room temperature for 15 h in a total volume of 25 μL using 5 U of EcoRI (Thermo Scientific, Waltham, MA, USA) and 5 U of MspI (or HpaII) (Thermo Scientific, USA). We used 1.5 U of T4 DNA ligase (Thermo Scientific, USA) for the ligation of 5 pmol of EcoRI and 50 pmol of MspI/HpaII double-stranded nucleotide adapters. The restriction ligation mixture was diluted with nuclease-free water to 2.5 ng/μL and used as a template in the pre-amplification reaction using primers that were complementary to the cores of the EcoRI and MspI/HpaII adapters (EcoRI + A and MspI/HpaII + T). Pre-selective and selective amplifications were performed with a MyCycler thermal cycler (Bio-Rad, Hercules, CA, USA). A 20 μL pre-amplification mixture contained 4 μL of the diluted restriction ligation mixture, 0.2 mM of dNTPs (Thermo Scientific, USA), 0.6 μM of each primer, 2 mM of MgCl_2_, 0.6 µM of both primers, 1X a Taq DNA polymerase buffer, and 0.5 U of a Taq DNA polymerase (Thermo Scientific, USA). After 1 min at 95 °C, DNA fragments were amplified for 20 cycles under the following conditions: 20 s at 94 °C, 30 s at 56 °C, and 2 min at 72 °C. After the final elongation step for 30 min at 60 °C, the pre-amplification product was diluted to 1:20 with nuclease-free water and used for selective amplification. This procedure was performed using the Eco-AAG, Mse/Hpa-TCC, Mse/Hpa-TAG, Eco-ACT, Mse/Hpa-TAC, Mse/Hpa-TAG, Eco-ACG, Mse/Hpa-TCC, and Mse/Hpa-TAG EcoRI-MspI/HpaII primer pairs. The Msp/Hpa primers were end-labelled with Hex or 6-Fam (Sigma-Aldrich, St. Louis, MO, USA). The final amplification mixture (20 μL) contained 3 μL of the diluted pre-amplification mixture, 0.2 mM of dNTPs (Thermo Scientific, USA), 2.5 mM of MgCl_2_, 0.05 μM of the EcoRI primer, 0.25 μM of the MspI/HpaII-labelled primer, 1X a Hot Start DNA polymerase buffer, and 0.5 U of Hot Start DNA Polymerase (Thermo Scientific, USA). After 2 min at 94 °C, the DNA fragments were amplified for 30 cycles under the following conditions: 20 s at 94 °C, 30 s at 66 °C for the first cycle followed by a decrease of 1 °C per cycle for 10 cycles, 56 °C for the remaining 20 cycles, and 2 min at 72 °C. A final extension step was performed for 30 min at 60 °C. The DNA fragments were detected using an automatic GeneScan ABI3130 apparatus from Macrogen Inc. (Korea). The presence or absence of fragments was scored on chromatograms using GeneMapper Genotyping Software 1.5. All fragments between 50 and 400 bp and passing the threshold of 100 RFU were scored. Rare fragments, e.g., those differing only in one individual, were deleted from the dataset. Furthermore, scored fragments were filtered in order to ensure reproducibility between runs. Fragments differing in average frequency for more than 0.15 between samples of the same population run in two different plates were eliminated from further analyses. To check for the error rate between runs, 24 samples were analysed twice using the Eco-ACT, Msp/Hpa-TAC, and Msp/Hpa-TAG primer combinations. The error rate was calculated for both the EM and EH sets as [40]:

$$\mathrm{error}=\frac{\mathrm{number}\mathrm{discordant}\mathrm{scores}\times 100}{\mathrm{number}\mathrm{of}\mathrm{scored}\mathrm{alleles}\times \mathrm{number}\mathrm{individuals}}$$

The error rate calculated using all of the scored fragments was 11.14% for the EcoRI-HpaII fragment set and 10.97% for the EcoRI-MspI fragment set. Using only the fragments retained after fragment-filtering the error rates were 4.03% and 4.75% for the EcoRI-HpaII and EcoRI-MspII fragment sets, respectively. 

MSAP fragments were analysed using the “msap” R package [41]. Every fragment was scored as follows: non-methylated state if present in both EcoRI-HpaII and EcoRI-MspI products (1/1); methylated state if present in either EcoRI-HpaII (1/0) or EcoRI-MspI (0/1) products (either internal cytosine methylation (0/1) or hemimethylation (1/0)); and hyper-methylation if fragment is absent from both EcoRI-HpaII and EcoRI-MspI products (0/0). Fragments were classified as “methylation-susceptible loci” (MSL) when the observed proportion of methylated scores (1/0, 0/1 and 0/0) exceeded a 5% threshold, and other fragments were considered “non-methylation loci” fragments (NML). NML fragments were further applied in analyses of genetics, and MSL fragments were further applied in analyses of epigenetic variability and differentiation. Only fragments showing a polymorphism, with at least two occurrences of each methylation state, were used for subsequent analyses.

For MSL fragments, the frequency of all methylation states across all MSL was calculated for each individual. Populations were statistically compared using an ANOVA followed by Tukey’s post hoc test using the “stats” R package (level of significance *p* ≤ 0.05), and the results are presented as mean ± SD. 

NML and MSL fragments were used for the analysis of genetic and epigenetic structures between populations with the Structure 2.3.4 software [42] using the following settings: burn-in period of 10,000, number of repeats of 1000, and no admixture. AMOVA (No. of Permutations = 1000), fixation index, and population pairwise FST values (No. of permutations for significance = 100, significance level = 0.05) were calculated for the NML and MSL fragment sets using Arlequin 3.5.2.2 [43]. Additionally, AMOVA and fixation indices were calculated using the MSL dataset on imposex levels as “populations”, which were grouped as low (1–4) and high (5–8) imposex level groups. Population genetic diversity indices were calculated using the Popgen software.

The GenAlEx 6.5 software was used to perform a principal coordinate analysis (PCoA) using the MSL fragments. PCoA graphs were generated using the epigenetic distance between individuals. An ANOVA and Tukey’s post hoc test were applied to the PCo1 and PCo2 scores in order to define significance in population separation using the “aov” and “TukeyHSD” functions, respectively, in R (*p* ≤ 0.05). 

The MSL and NML fragment sets were used to analyse the fragments under selection (outliers) using the Arlequin 3.5.2.2., Mcheza [44], and BayeScan [45] software. Loci under selection in Arlequin were determined using a hierarchical island model (No. of simulated groups: 10; No. of simulated demes per groups: 100; No. of coalescent simulations performed: 20,000), and they were considered outliers if they were above the 1st quantile. Mcheza uses a Dfdist, a modification of Fdist [46], which allows for dominant markers and implements the method of Zhivotovsky [47] to estimate allele frequencies. Mcheza was run with 10,000 simulations with the “neutral FST” and “force mean FST” options, and it selected loci outside the upper tail of the 99% confidence interval. BayeScan analysis exploited a Bayesian inference method to directly estimate the posterior probability (PP) for each locus under selection. Analysis was conducted with 5000 iterations, a thinning interval of 10, and a burn-in length of 50,000. The number of pilot runs was kept at 20, with a length of 5000 each. Outliers were defined at a threshold of q ≤ 0.05. 

Using the population pairwise FST values obtained through Arlequin and geographic distances between populations, the Mantel test was performed using the “mantel.rtest” function in the “ade4” R package to check for isolation by distance (IBD).

## 3. Results

### 3.1. OTCs in Snail Tissue

The amount of accumulated TBT in the whole murex tissue from two lowly polluted sites was significantly lower in comparison to the amount recorded at the highly polluted sites (Figure 2A). The mean TBT concentrations at the lowly polluted sites of Čiovo and Ston were 1.8 and 2.6 ng Sn g^−1^, respectively. At the intermediately polluted site of Marina, the mean TBT concentration was 10.7 ng Sn g^−1^, and at the four highly polluted sites, the mean TBT concentrations ranged from 21.4 to 43.1 ng Sn g^−1^. The amount of accumulated TBT in specimens with low imposex levels (1–4) was significantly lower in comparison to the amount recorded in snails with higher imposex levels (5–8) (Figure 2B). The mean values of accumulated TBT in snails with imposex levels of 1–3 were between 0.7 and 2.0 ng Sn g^−1^, whereas in those with imposex levels of 5–8, the mean values ranged between 12.3 and 29.0 ng Sn g^−1^. The accumulated DBT followed a similar trend. At the Čiovo and Ston sites, the mean DBT concentrations were 2.9 and 1.4 ng Sn g^−1^, respectively. At the Marina site, the mean DBT concentration was 2.5, whereas at the highly polluted sites, the mean DBT concentrations ranged from 29.3 to 81.3 ng Sn g^−1^.

### 3.2. Phenotypic Responses

#### 3.2.1. Imposex

**Figure 3:**
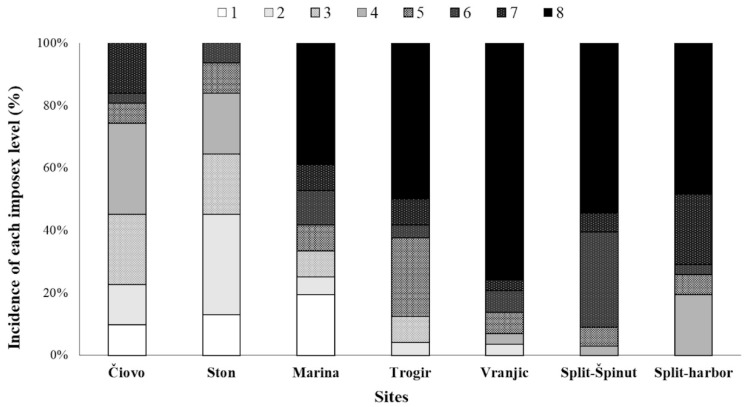
Percentage of banded murex (*H. trunculus*) females collected in the Adriatic Sea with specific imposex levels per sampling site. 1–8: imposex levels (from normal females (1) to the highest imposex level (8)).

At the sites with a low pollution intensity, Čiovo and Ston, the majority of females (74.2% and 83.9%, respectively) had imposex levels in the lower range (1–4) and no individuals with an imposex level of 8 were recorded. At the intermediately polluted site of Marina, 33.3% of females had lower imposex levels (1–4) and 38.9% of females had the highest imposex level (8). At the four highly polluted sites, none of the examined females had the lowest imposex level (1) and between 80.6% and 97% of females had higher imposex levels (5–8) (Figure 3).

Both TBT and DNA methylation were found to be significant predictors of imposex, as shown using the ordinal logit regression model (Table 2). Based on the model output, we can expect that for a one unit increase in TBT, an 0.03 increase in the expected value of imposex can be predicted on a log odds scale (Table 2A). For DNA methylation, the model showed that the decrease in DNA methylation increased the likelihood of having a higher imposex level (Table 2B).

#### 3.2.2. Biomarkers

Although the biomarker results showed some population-specific responses, they did not indicate the separation of the sites according to the TBT pollution level. The PCA only revealed the significant separation of the intermediately polluted site, Marina, from the lowly and highly polluted sites in PC1 (Figure S1, Supporting Information). Altogether, 53.48% of the variance of the biomarker data for different populations was explained by PC1 and PC2. 

#### 3.2.3. Morphometry

The PCA of *H. trunculus* morphometric measures showed the significant separation of female snails originating from highly polluted sites, Trogir and Split-Špinut, from the other sites in PC1 (Figure S2A, Supporting Information). A significant separation of a lowly polluted site, Ston, from the intermediately and highly polluted sites in PC2 for females and in PC1 and PC2 for males was also observed (Figure S2A,B, Supporting Information). Two dominant PCA components (PC1 and PC2) explained 69.17% and 75.83% of the variance for females and males, respectively. There was no correlation between the morphometric and geographic distance matrices for either males or females according to the Mantel test. Likewise, no correlation was revealed between morphometric and epigenetic or genetic distances.

The total mass of the soft tissue (WM) was significantly different between sites of low pollution intensity, Čiovo and Ston, and intermediately and highly polluted sites, apart from Vranjic (Figure S3A, Supporting Information). Moreover, females with low imposex levels (1–4) had significantly lower WM values in comparison to females with high imposex levels (5–8) (Figure S3B, Supporting Information). The one-way ANOVA was significant in predicting the snail WM by imposex level (F-statistic of 2.277 on 7 and 101 DF; *p*-value of 0.034). Using linear regression, both TBT accumulation in snail tissue and DNA methylation were significant predictors of snail mass (F-statistic of 7.766 on 1 and 55 DF; *p*-value of 0.007 for TBT and F-statistic of 4.466 on 1 and 195 DF; *p*-value of 0.036 for DNA methylation). In general, the increase in snail mass was linked to a higher imposex level, higher TBT accumulation, and decrease in DNA methylation.

### 3.3. DNA Methylation

After fragment-filtering, 568 fragments were retained. We determined 355 polymorphic MSL and 43 polymorphic NML using the “msap” function in R. Considering all methylation states (internal and external cytosine methylation and hypermethylation), individuals from the clean site of Čiovo had significantly higher levels of DNA methylation than individuals from the polluted sites of Trogir, Vranjic and Split-Špinut (Figure 2C). Overall, individuals with low (1–4) imposex levels had significantly higher levels of DNA methylation than individuals with high (5–8) imposex levels (Figure 2D). 

Neither polymorphic MSL nor NML fragments revealed any obvious epigenetic or genetic structures among the analysed populations using the Structure software. The results of the Mantel test confirmed a lack of isolation-by-distance pattern in either epigenetic (r = 0.022; *p* = 0.16) or genetic (r = −0.019; *p* = 0.40) differentiation. 

However, the AMOVA of the polymorphic MSL and NML fragments revealed a small but significant portion of variation among populations in both datasets (Table 3). The epigenetic differentiation among populations was significant (FST = 0.0258; *p* < 0.001) and considerably stronger than the genetic differentiation (FST = 0.0096; *p* < 0.001) (Table 3). The variation among population groups pooled based on the imposex levels was not significant.

The population genetic diversity was observed to be lower than the population epigenetic diversity (Table S2), as shown by the effective number of alleles, Nei’s gene diversity, and Shannon’s information index. Populations from intermediately and highly polluted sites exhibited higher epigenetic diversity levels than the two populations from the lowly polluted sites, Čiovo and Ston. Linear regression models using individual TBT accumulation in snail tissue as a predictor and individual DNA methylation were not significant (F-statistic of 2.415 on 1 and 62 DF; *p*-value of 0.1253). However, the mean population TBT burden was found to be a significant predictor of mean population DNA methylation (F-statistic of 9.867 on 1 and 5 DF; *p*-value of 0.02563; adjusted R-squared of 0.5964).

**Figure 4:**
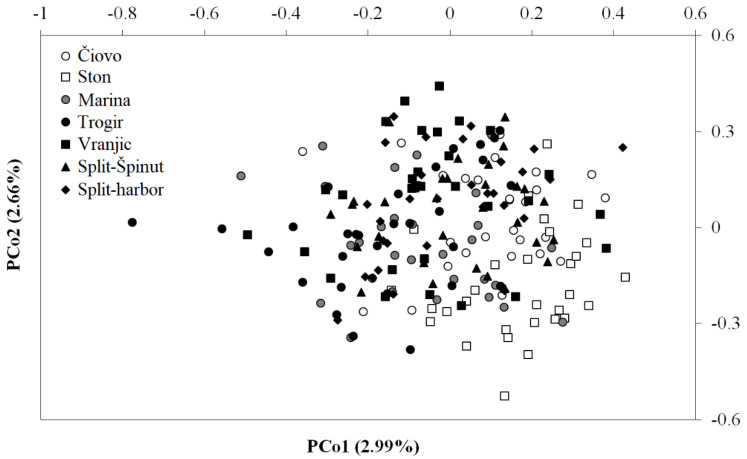
Principal coordinate analysis of epigenetic (MSL) fragments obtained from banded murex (*H. trunculus*) individuals collected in the Adriatic Sea. Colour codes relate to site pollution intensity: white—lowly polluted sites (Čiovo and Ston); grey—intermediately polluted sites (Marina); black—highly polluted sites (Trogir, Vranjic, Split-Špinut, and Split-harbour). In PCo1, lowly polluted sites were significantly separated from intermediately and highly polluted sites. In PCo2, lowly and intermediately polluted sites were significantly separated from highly polluted sites; ANOVA and Tukey’s post hoc test, *p* ≤ 0.05.

The principal coordinate analysis (PCoA) based on individual pairwise genetic distances using the MSL dataset explained 5.65% of variation in first two coordinates and showed a significant separation of sites based on pollution intensity. Sites with a low pollution intensity were significantly separated along the PCo1 coordinate from the intermediately and highly polluted sites (Figure 4). This pattern was not observed for the NML dataset.

For epigenetic (MSL) fragments, the pairwise FST differentiation between populations ranged from 0.002 to 0.61 and was significant between almost all population pairs except between the high-pollution sites of Split-harbour and Trogir and between Split-harbour and Vranjic (Table 4). For genetic (NML) fragments, the pairwise FST differentiation ranged from 0 to 0.028, and it was only significant in few cases (Table 4).

Using the MSL dataset, seven outliers were detected (six using Arlequin, three using Mcheza, and none using BayeScan). A total of two outliers overlapped between Arlequin-and Mcheza-detected outliers (Table S3A). Using the NML dataset, only one outlier was detected with Arlequin and Mcheza (Table S3B).

Overall, analyses showed higher degrees of epigenomic than genomic within-population diversity and among-population differentiation. No evidence was found that population differentiation stemmed from geographic distances, and PCoA indicated some effect of pollution status on epigenetic divergence.

## 4. Discussion

The results of imposex occurrence in murex populations at sites of high marine traffic activities along the Croatian coast presented here are rather alarming, as we recorded that around 50% or more of female snails inhabiting highly polluted sites exhibited the highest imposex level, indicating possible sterility. This is in agreement with other recent data that point to the persistence and constant input of TBT in ports and marinas along the Croatian coast, as well as its notable impact on the reproductive fitness of murex populations [10,27]. 

The TBT-related phenotypic response was further evident in terms of increases in the soft tissue mass (WM) of murex snails, which followed increases in imposex level and bioaccumulated TBT in females. TBT is a known obesogenic factor that can modulate changes in the type and number of generated adipocyte cells [24]. Both in vitro and in vivo studies have linked TBT exposure to altered lipid metabolism and/or adipocyte differentiation in various vertebrates and invertebrates, often pointing to the underlying involvement of the PPARγ and RXR pathways (reviewed in [48]). Recently, it has also been shown that TBT induced changes in lipid metabolism in rotifers thorough the modulation of RXR signalling [49]. Interestingly, it was also observed that the TBT-induced adipocyte differentiation was accompanied by a decrease in global DNA methylation, indicating the potential of underlying epigenetic mechanisms [21]. Even though relative WM is driven by many environmental factors, most geographical sites sampled in this study covered very limited spatial areas in which these factors were not expected to significantly vary. Our analyses therefore suggest a relationship between murex imposex incidence, increases in body mass, and global DNA methylation across individual data. Contrary to our findings, a 10% decrease in WM was previously noted for native females with imposex in the *Odontocymbiola magellanica* marine gastropod [50].

Epigenetic modifications, such as DNA methylation, have been argued as important players in an organism’s response to altered environmental conditions. In our study, higher incidences of imposex and accumulated TBT in individuals inhabiting ports and marinas were accompanied by decreases in genome-wide DNA methylation. We observed significant effects of both TBT accumulation and DNA methylation on imposex advancement. Furthermore, population-level, genome-wide DNA demethylation was correlated with TBT tissue burden, indicating undirected and stochastic TBT epigenetic alterations that are likely to drive maladaptive biological consequences, such as the observed decrease in reproductive fitness.

A decrease in the level of DNA methylation as a result of TBT exposure has also been previously reported in laboratories at both the genome and gene-specific levels. DNA demethylation was observed following the in vitro TBT exposure of preadipocyte mouse3T3-L1 cells [21]. In stromal mouse cells, TBT caused a decrease in DNA methylation in the promoter/enhancer region of the Fabp4 gene, which was followed by an increase in its expression [20]. Global DNA demethylation was also observed following in vivo TBT exposure in the liver of marble rockfish (*Sebastiscus marmoratus*) and in gastropods (*Tritia mutabilis*) exposed during embryonic development [22]. Moreover, in *T. mutabilis,* a simultaneous decrease in DNA-methyltransferase I (DNMT1) expression pointed towards global hypomethylation underlying altered RXR expression. However, we cannot fully confirm the causality of DNA methylation in the cascade of TBT-induced phenotypic responses in the studied imposex-affected murex. The link between epigenetic change and specific phenotypes might indicate that these changes are simply correlated and do not necessarily mean that the epigenetic variant is causative of the phenotype [2]. To fully understand the function of DNA methylation in development of specific phenotypic responses, the role of DNA methylation at specific sites of interest needs to be interrogated [2]. Therefore, focused studies examining the DNA methylation of genes involved in animal reproductive fitness and growth should be prioritised. However, the mechanisms of TBT-induced imposex development are still not fully understood, and the involvement of many genes and pathways have been proposed [51,52]. Previously, imposex occurrence was attributed to either the inhibition of aromatase activity, which was followed by an increase in free testosterone in females or neurotoxic TBT properties causing abnormal neurohormone secretion [8,53,54]. However, through the last decade, accumulating evidence has linked imposex occurrence to the modulation of the nuclear retinoid X receptor (RXR) [7,22,55,56,57]. TBT acts as a ligand and activates molluscan RXR [58]. It has also been shown that TBT-induced imposex is, alongside RXR, mediated by the genes of the peroxisome proliferator-activated receptor (PPAR) pathways [52]. Interestingly, the same nuclear receptors are involved in TBT-induced adipocyte differentiation and the onset of obesity [21,58]. Thus, we suggest that further studies should focus on examining the DNA methylation of genes involved in PPAR and/or RXR activation in order to fully elucidate the role and extent of epigenetic regulation in imposex development and animal growth.

Despite the lack of isolation by distance, showing no significant influence of geographic distance on genetic or epigenetic divergence, the studied murex populations were significantly genetically and epigenetically differentiated. Notably, the extent of epigenetic differentiation observed among the snail populations was considerably stronger than the genetic differentiation. A low genetic differentiation implies that at this geographic scale the gene flow among populations is strong enough to prevent substantial genetic divergence. The relatively low resolution of genetic markers used in this study prevented us from conducting the deeper evaluation of population genetic adaptation. 

Ecological divergence is increasingly recognised as a major driver of misalignment between patterns of epigenetic and genetic population differentiation [59]. Numerous previous studies have pointed to the involvement of epigenetic mechanisms in biological responses toward the environment in animal populations lacking strong genetic differentiation. Populations of the sandhopper *Talorchestia capensis* exhibited greater epigenetic than genetic variability, and this was attributed to the role of epigenetics in response to local environmental conditions [3]. The effect of the environment on populations’ epigenetic profiles was further documented in two bivalve species that exhibited genome-wide demethylation linked to environmental stress, e.g., salinity and pollution [60], and in clonal populations of freshwater snail *Potamopyrgus antipodarum* for which epigenetic divergence associated with adaptive phenotypes in contrasting environments was observed [61]. Given TBT’s strong potential to alter DNA methylation patterns and the observed biological effects of bioaccumulated TBT at the fitness level, it is quite plausible that the observed epigenetic differentiation in *H. trunculus* stems from differences in local environmental conditions, of which pollution is one major factor. 

The higher epigenetic differentiation in comparison to genetic differentiation in our study was further supported by the detected outlier loci in our datasets. Whereas only one outlier was recorded in the genetic loci, seven outliers were defined using the genome scan approach in the epigenetic dataset. The detected outliers remained anonymous, and to this end, their potential role in organism adaptation to local environmental conditions cannot be discussed. The detection of candidate loci that are potentially subject to selection is a widely applied approach using genome-wide genetic data, and it is being increasingly applied for epigenetic markers as well [62,63,64]. It has been postulated that the detection of epigenetic outliers is hindered by several mechanisms. Epigenetic variation may be dependent on genetic variation, and it might be hard to evaluate whether the epigenetic or underlying genetic state is under selection [65]. Furthermore, epigenetic variation can be caused by randomly occurring epimutations or induced by the environment, which implicates different selection processes [59,66]. Additionally, the degree of heritability of epigenetic variation (ranging from few to several generations) can have an impact on the selective outcome, as adaptive traits are only selected by natural selection if they are heritable [65]. However, evidence from natural populations suggests that epigenetic stability is a selective trait [67,68,69].

Despite the significant differences in TBT accumulation and imposex levels between the sites of low and high marine activity, no clear differentiation in snail biomarker responses that could be linked to the impact of TBT (or to the overall marine traffic pollution) was recorded in our study. Previous laboratory exposure experiments indicated that TBT acts as an inducer of oxidative stress in gastropods, fish and mammals [70,71,72]. The onset of oxidative stress in native populations of the *Buccinanops globulosus* gastropod with high imposex occurrence inhabiting harbours has been attributed to TBT and other contaminants such as trace metals and polyaromatic hydrocarbons [73]. However, native populations are exposed to TBT in a mixture of various pollutants, which might have opposing effects on organisms’ overall oxidative status, hence preluding the straightforward disentanglement of TBT-specific oxidative responses. Furthermore, the lack of a clear biomarker response could be due to acclimatization or adaptation to constant pollutant input or to other environmental drivers often observed in native populations [74,75]. 

The lack of correlation of morphological distances with either geographic or genetic ones indicates the effect of local environmental conditions on morphological differentiation and the probable involvement of phenotypic plasticity. For instance, females from highly polluted sites, Trogir and Split-Špinut, were significantly separated from other sites in PCA. Female snails from those sites also had the highest level of accumulated TBT in their tissue. Several studies previously reported that TBT exposure can affect shell shape in gastropods [50,76,77]. We also observed that the female and male snails from the lowly polluted site of Ston were significantly separated from the other sites. Thus, as the snail morphological divergence could not be linked to the geographic distances between populations, the morphological differentiation of populations can presumably be driven by the specific local environmental conditions, including pollution loads.

In conclusion, the results of the present study highlight the potential of epigenetic studies to unravel pollution-induced alterations in wild populations. DNA methylation might be crucial for explaining specific phenotypic developmental alterations such as imposex; however, further studies are necessary to determine the exact causative epigenetic mechanisms. 

## Figures and Tables

**Table 1:** Description of the imposex levels assessed in this study, according to [26,29].

	Imposex Stage	Description
1	0	Normal females
2	1	Presence of a tiny penis behind the right ocular tentacle
3	2	Both penis and penis duct are present
4	3	Development of a penis and a short vas deferens
5	4	Presence of a penis and a vas deferens which reaches the vagina
6	4.3	The vas deferens passes the vaginal opening
7	4.7	The vas deferens runs along 30% of the capsule gland
8	5	No vulva is visible, and the capsule gland may be split

**Table 2:** Summary of the ordinal logit regression model using TBT (A) and DNA methylation (B) as predictors of imposex level in banded murex (*H. trunculus*) individuals collected in the Adriatic Sea. Coefficient value and estimates for the intercepts (cutpoints) including standard error (SE), t-value and *p*-value (<0.05 *, <0.01 **, and <0.001 ***). OR: odds ratio; CI: confidence interval; AIC: Akaike information criterion.

(A)		Value	SE	t-Value	*p*-Value	OR	CI	AIC
**TBT**	**Coefficient**	0.03	0.01	2.47	0.01345 *	1.03	1.01–1.05	236.49
1|2	Intercepts	−2.32	0.53	−4.35	0.00001 ***			
2|3		−1.53	0.41	−3.77	0.00017 ***			
3|4		−1.26	0.38	−3.32	0.00091 ***			
4|5		−0.62	0.34	−1.83	0.06750			
5|6		0.03	0.33	0.10	0.92236			
6|7		0.73	0.34	2.15	0.03153 *			
7|8		1.07	0.35	3.06	0.00220 **			
**(B)**		**Value**	**SE**	**t-Value**	***p*-Value**	**OR**	**CI**	**AIC**
**Methylation**	**Coefficient**	−47.17	12.69	−3.72	0.00020 ***	3.28 × 10^−21^	2.36 × 10^−32^–1.17 × 10^−10^	406.93
1|2	Intercepts	−45.00	11.30	−3.98	0.00007 ***			
2|3		−43.95	11.29	−3.89	0.00010 ***			
3|4		−43.36	11.28	−3.84	0.00012 ***			
4|5		−42.80	11.26	−3.80	0.00014 ***			
5|6		−42.37	11.24	−3.77	0.00016 ***			
6|7		−41.85	11.22	−3.73	0.00019 ***			
7|8		−41.44	11.21	−3.70	0.00022 ***			

**Table 3:** Population-level AMOVA of epigenetic (MSL) and genetic (NML) fragments obtained from banded murex (*H. trunculus*) individuals collected in the Adriatic Sea.

	EPIGENETIC (MSL)		GENETIC (NML)	
**Source of Variation**	**Percentage Variation**	Fixation Index (* *p* < 0.05)	**Percentage Variation**	Fixation Index (* *p* < 0.05)
Among populations	2.58	F_ST_: 0.02581 *	0.96	F_ST_: 0.00964 *
Within populations	97.42		99.04	

* *p* < 0.05.

**Table 4:** Pairwise epigenetic (MSL fragments, below diagonal) and genetic (NML fragments, above diagonal) differentiation (AMOVA-based F_ST_) among populations of banded murex (*H. trunculus*) collected in the Adriatic Sea. Star denotes the statistical significance of F_ST_ value between pairs of populations (* *p* < 0.05).

	Čiovo	Ston	Marina	Trogir	Vranjic	Split-Špinut	Split-Harbour
Čiovo	-	0.006	0.025 *	0	0.005	0.017 *	0.024 *
Ston	0.036 *	-	0.028 *	0.008	0.014 *	0	0.018
Marina	0.048 *	0.061 *	-	0.028 *	0.005	0.014	0.001
Trogir	0.016 *	0.015 *	0.028 *	-	0	0.016 *	0.014
Vranjic	0.019 *	0.030 *	0.056 *	0.011 *	-	0.001	0
Split-Špinut	0.026 *	0.015 *	0.049 *	0.015 *	0.009 *	-	0
Split-Harbour	0.028 *	0.022 *	0.049 *	0.002	0.003	0.012*	-

## Data Availability

All data needed to evaluate the conclusions in the paper are present in the paper and/or the Supplementary Materials.

## Supplementary Materials

The following Supporting Information can be downloaded at: https://www.mdpi.com/article/10.3390/toxics11030276/s1.

## References

[1] Dimond J.L., Roberts S.B. (2016). Germline DNA Methylation in Reef Corals: Patterns and Potential Roles in Response to Environmental Change. Mol. Ecol..

[2] Duncan E.J., Gluckman P.D., Dearden P.K. (2014). Epigenetics, Plasticity, and Evolution: How Do We Link Epigenetic Change to Phenotype?. J. Exp. Zool. Part B Mol. Dev. Evol..

[3] Baldanzi S., Costera E., Marinas D.I., Cruces L., Secondary C.A., Author C., Baldanzi S., Watson R., Mcquaid C., Gouws G. (2017). Evolutionary Ecology Epigenetic Variation among Natural Populations of the South African Sandhopper *Talorchestia capensis*. Evol. Ecol..

[4] Jablonka E., Raz G. (2009). Transgenerational Epigenetic Inheritance: Prevalence, Mechanisms, and Implications for the Study of Heredity and Evolution. Q. Rev. Biol..

[5] Schrey A.W., Alvarez M., Foust C.M., Kilvitis H.J., Lee J.D., Liebl A.L., Martin L.B., Richards C.L., Robertson M. (2013). Ecological Epigenetics: Beyond MS-AFLP. Integr. Comp. Biol..

[6] Abidli S., Castro L.F.C., Lahbib Y., Reis-Henriques M.A., Trigui El Menif N., Santos M.M. (2013). Imposex Development in *Hexaplex trunculus* (Gastropoda: Caenogastropoda) Involves Changes in the Transcription Levels of the Retinoid X Receptor (RXR). Chemosphere.

[7] Lima D., Reis-Henriques M.A., Silva R., Santos A.I., Filipe C., Castro L., Santos M.M. (2011). Tributyltin-Induced Imposex in Marine Gastropods Involves Tissue-Specific Modulation of the Retinoid X Receptor. Aquat. Toxicol..

[8] Abidli S., Santos M.M.H., Lahbib Y., Castro L.F.C., Reis-Henriques M.A., Trigui El Menif N. (2012). Tributyltin (TBT) Effects on *Hexaplex trunculus* and *Bolinus brandaris* (Gastropoda: Muricidae): Imposex Induction and Sex Hormone Levels Insights. Ecol. Indic..

[9] Bryan G.W., Gibbs P.E., Hummerstone L.G., Burt G.R. (1986). The Decline of the Gastropod *Nucella lapillus* Around South-West England: Evidence for the Effect of Tributyltin from Antifouling Paints. J. Mar. Biol. Assoc. U. K..

[10] Furdek M., Vahčič M., Ščančar J., Milačič R., Kniewald G., Mikac N. (2012). Organotin Compounds in Seawater and *Mytilus galloprovincialis* Mussels along the Croatian Adriatic Coast. Mar. Pollut. Bull..

[11] Pougnet F., Schäfer J., Dutruch L., Garnier C., Tessier E., Dang D.H., Lanceleur L., Mullot J.-U., Lenoble V., Blanc G. (2014). Sources and Historical Record of Tin and Butyl-Tin Species in a Mediterranean Bay (Toulon Bay, France). Environ. Sci. Pollut. Res..

[12] Langston W.J., Pope N.D., Davey M., Langston K.M., O’ Hara S.C.M., Gibbs P.E., Pascoe P.L. (2015). Recovery from TBT Pollution in English Channel Environments: A Problem Solved?. Mar. Pollut. Bull..

[13] Furdek M., Mikac N., Bueno M., Tessier E., Cavalheiro J., Monperrus M. (2016). Organotin Persistence in Contaminated Marine Sediments and Porewaters: In Situ Degradation Study Using Species-Specific Stable Isotopic Tracers. J. Hazard. Mater..

[14] Harrison T.D., Gilmour G., McNeill M.T., Armour N., McIlroy L. (2020). Survey of Imposex in *Nucella lapillus* as an Indicator of Tributyltin Pollution in Northern Irish Coastal Waters, 2004 to 2017. Mar. Pollut. Bull..

[15] Formalewicz M.M., Rampazzo F., Noventa S., Gion C., Petranich E., Crosera M., Covelli S., Faganeli J., Berto D. (2019). Organotin Compounds in Touristic Marinas of the Northern Adriatic Sea: Occurrence, Speciation and Potential Recycling at the Sediment-Water Interface. Environ. Sci. Pollut. Res..

[16] Nicolaus E.E.M., Barry J. (2015). Imposex in the Dogwhelk (*Nucella lapillus*): 22-Year Monitoring around England and Wales. Environ. Monit. Assess..

[17] Ruiz J.M., Carro B., Albaina N., Couceiro L., Míguez A., Quintela M., Barreiro R. (2017). Bi-Species Imposex Monitoring in Galicia (NW Spain) Shows Contrasting Achievement of the OSPAR Ecological Quality Objective for TBT. Mar. Pollut. Bull..

[18] Sousa A.C.A., Pastorinho M.R., Takahashi S., Tanabe S. (2014). History on Organotin Compounds, from Snails to Humans. Environ. Chem. Lett..

[19] Wang Y., Wang C., Zhang J., Chen Y., Zuo Z. (2009). DNA Hypomethylation Induced by Tributyltin, Triphenyltin, and a Mixture of These in *Sebastiscus marmoratus* Liver. Aquat. Toxicol..

[20] Kirchner S., Kieu T., Chow C., Casey S., Blumberg B. (2010). Prenatal Exposure to the Environmental Obesogen Tributyltin Predisposes Multipotent Stem Cells to Become Adipocytes. Mol. Endocrinol..

[21] Bastos Sales L., Kamstra J.H., Cenijn P.H., van Rijt L.S., Hamers T., Legler J. (2013). Effects of Endocrine Disrupting Chemicals on in Vitro Global DNA Methylation and Adipocyte Differentiation. Toxicol. Vitr..

[22] Cocci P., Mosconi G., Palermo F.A. (2021). Effects of Tributyltin on Retinoid X Receptor Gene Expression and Global DNA Methylation during Intracapsular Development of the Gastropod *Tritia mutabilis* (Linnaeus, 1758). Environ. Toxicol. Pharmacol..

[23] Chamorro-Garcia R., Diaz-Castillo C., Shoucri B.M., Käch H., Leavitt R., Shioda T., Blumberg B. (2017). Ancestral Perinatal Obesogen Exposure Results in a Transgenerational Thrifty Phenotype in Mice. Nat. Commun..

[24] Grün F., Blumberg B. (2006). Environmental Obesogens: Organotins and Endocrine Disruption via Nuclear Receptor Signaling. Endocrinology.

[25] Suarez-Ulloa V., Gonzalez-Romero R., Eirin-Lopez J.M. (2015). Environmental Epigenetics: A Promising Venue for Developing next-Generation Pollution Biomonitoring Tools in Marine Invertebrates. Mar. Pollut. Bull..

[26] Axiak V., Vella A.J., Micallef D., Chircop P., Mintoff B. (1995). Imposex in *Hexaplex trunculus* (Gastropoda: Muricidae): First Results from Biomonitoring of Tributyltin Contamination in the Mediterranean. Mar. Biol..

[27] Erdelez A., Furdek Turk M., Štambuk A., Župan I., Peharda M. (2017). Ecological Quality Status of the Adriatic Coastal Waters Evaluated by the Organotin Pollution Biomonitoring. Mar. Pollut. Bull..

[28] Waite M.E., Waldock M.J., Thain J.E., Smith D.J., Milton S.M. (1991). Reductions in TBT Concentrations in UK Estuaries Following Legislation in 1986 and 1987. Mar. Environ. Res..

[29] Terlizzi A., Geraci S., Gibbs P.E. (1999). Tributyltin (TBT)-induced Imposex in the Neogastropod *Hexaplex trunculus* in Italian Coastal Waters: Morphological Aspects and Ecological Implications. Ital. J. Zool..

[30] Santos J.A., Galante-Oliveira S., Barroso C. (2011). An Innovative Statistical Approach for Analysing Non-Continuous Variables in Environmental Monitoring: Assessing Temporal Trends of TBT Pollution. J. Environ. Monit..

[31] Aebi H. (1984). Catalase in Vitro. Methods Enzymol..

[32] Ramos-Martinez J.I., Bartolomé T.R., Pernas R.V. (1983). Purification and Properties of Glutathione Reductase from Hepatopancreas of *Mytilus edulis* L. Comp. Biochem. Physiol. Part B Comp. Biochem..

[33] Habig W.H., Pabst M.J., Jakoby W.B. (1974). Glutathione S-Transferases. The First Enzymatic Step in Mercapturic Acid Formation. J. Biol. Chem..

[34] Ellman G.L., Courtney K.D., Andres V., Featherstone R.M. (1961). A New and Rapid Colorimetric Determination of Acetylcholinesterase Activity. Biochem. Pharmacol..

[35] Levine R.L., Williams J.A., Stadtman E.R., Shacter E. (1994). Carbonyl Assays for Determination of Oxidatively Modified Proteins. Methods Enzymol..

[36] Buege J.A., Aust S.D. (1978). Microsomal Lipid Peroxidation. Methods Enzymol..

[37] Bradford M.M. (1976). A Rapid and Sensitive Method for the Quantitation of Microgram Quantities of Protein Utilizing the Principle of Protein-Dye Binding. Anal. Biochem..

[38] Chiavarini S., Massanisso P., Nicolai P., Nobili C., Morabito R. (2003). Butyltins Concentration Levels and Imposex Occurrence in Snails from the Sicilian Coasts (Italy). Chemosphere.

[39] Xu M., Li X., Korban S.S. (2000). AFLP-Based Detection of DNA Methylation. Plant Mol. Biol. Report..

[40] Herrera C.M., Bazaga P. (2009). Quantifying the Genetic Component of Phenotypic Variation in Unpedigreed Wild Plants: Tailoring Genomic Scan for within-Population Use. Mol. Ecol..

[41] Pérez-Figueroa a. (2013). Msap: A Tool for the Statistical Analysis of Methylation-Sensitive Amplified Polymorphism Data. Mol. Ecol. Resour..

[42] Pritchard J.K., Stephens M., Donnelly P. (2000). Inference of Population Structure Using Multilocus Genotype Data. Genetics.

[43] Excoffier L., Lischer H.E.L. (2010). Arlequin Suite Ver 3.5: A New Series of Programs to Perform Population Genetics Analyses under Linux and Windows. Mol. Ecol. Resour..

[44] Antao T., Beaumont M.A. (2011). Mcheza: A Workbench to Detect Selection Using Dominant Markers. Bioinformatics.

[45] Foll M., Gaggiotti O. (2008). A Genome-Scan Method to Identify Selected Loci Appropriate for Both Dominant and Codominant Markers: A Bayesian Perspective. Genetics.

[46] Beaumont M.A., Nichols R.A. (1996). Evaluating Loci for Use in the Genetic Analysis of Population Structure. Proc. R. Soc. B Biol. Sci..

[47] Zhivotovsky L.A. (1999). Estimating Population Structure in Diploids with Multilocus Dominant DNA Markers. Mol. Ecol..

[48] Egusquiza R.J., Blumberg B. (2020). Environmental Obesogens and Their Impact on Susceptibility to Obesity: New Mechanisms and Chemicals. Endocrinology.

[49] Lee M.-C., Fonseca E., Park J.C., Yoon D.-S., Choi H., Kim M., Han J., Cho H.-S., Shin K.-H., Santos M.L. (2019). Tributyltin Affects Retinoid X Receptor-Mediated Lipid Metabolism in the Marine Rotifer *Brachionus koreanus*. Environ. Sci. Technol..

[50] Bigatti G., Carranza A. (2007). Phenotypic Variability Associated with the Occurrence of Imposex in *Odontocymbiola magellanica* from Golfo Nuevo, Patagonia. J. Mar. Biol. Assoc. U. K..

[51] Langston W.J. (2020). Endocrine Disruption and Altered Sexual Development in Aquatic Organisms: An Invertebrate Perspective. J. Mar. Biol. Assoc. U. K..

[52] Pascoal S., Carvalho G., Vasieva O., Hughes R., Cossins A., Fang Y., Ashelford K., Olohan L., Barroso C., Mendo S. (2013). Transcriptomics and in Vivo Tests Reveal Novel Mechanisms Underlying Endocrine Disruption in an Ecological Sentinel, *Nucella lapillus*. Mol. Ecol..

[53] Bettin C., Oehlmann J., Stroben E. (1996). TBT-Induced Imposex in Marine Neogastropods Is Mediated by an Increasing Androgen Level. Helgoländer Meeresunters..

[54] Oberdörster E., McClellan-Green P. (2002). Mechanisms of Imposex Induction in the Mud Snail, *Ilyanassa obsoleta*: TBT as a Neurotoxin and Aromatase Inhibitor. Mar. Environ. Res..

[55] Castro L., Lima D., Machado A., Melo C., Hiromori Y., Nishikawa J., Nakanishi T., Reis-Henriques M.A., Santos M.M. (2007). Imposex Induction Is Mediated through the Retinoid X Receptor Signalling Pathway in the Neogastropod *Nucella lapillus*. Aquat. Toxicol..

[56] Stange D., Sieratowicz A., Oehlmann J. (2012). Imposex Development in Nucella Lapillus—Evidence for the Involvement of Retinoid X Receptor and Androgen Signalling Pathways in Vivo. Aquat. Toxicol..

[57] Giulianelli S., Primost M.A., Lanari C., Bigatti G. (2020). RXR Expression in Marine Gastropods with Different Sensitivity to Imposex Development. Sci. Rep..

[58] Lyssimachou A., Santos J.G., André A., Soares J., Lima D., Guimarães L., Almeida C.M.R., Teixeira C., Castro L.F.C., Santos M.M. (2015). The Mammalian “Obesogen” Tributyltin Targets Hepatic Triglyceride Accumulation and the Transcriptional Regulation of Lipid Metabolism in the Liver and Brain of Zebrafish. PLoS ONE.

[59] Rey O., Eizaguirre C., Angers B., Baltazar-Soares M., Sagonas K., Prunier J.G., Blanchet S. (2020). Linking Epigenetics and Biological Conservation: Towards a Conservation Epigenetics Perspective. Funct. Ecol..

[60] Ardura A., Clusa L., Zaiko A., Garcia-Vazquez E., Miralles L. (2018). Stress Related Epigenetic Changes May Explain Opportunistic Success in Biological Invasions in Antipode Mussels. Sci. Rep..

[61] Thorson J.L.M., Smithson M., Beck D., Sadler-Riggleman I., Nilsson E., Dybdahl M., Skinner M.K. (2017). Epigenetics and Adaptive Phenotypic Variation between Habitats in an Asexual Snail. Sci. Rep..

[62] Wenzel M.A., Piertney S.B. (2014). Fine-Scale Population Epigenetic Structure in Relation to Gastro-Intestinal Parasite Load in Red Grouse (*Lagopus lagopus scotica*). Mol. Ecol..

[63] Wogan G.O.U., Yuan M.L., Mahler D.L., Wang I.J. (2020). Genome-Wide Epigenetic Isolation by Environment in a Widespread Anolis Lizard. Mol. Ecol..

[64] Caizergues A.E., Le Luyer J., Grégoire A., Szulkin M., Senar J.C., Charmantier A., Perrier C. (2022). Epigenetics and the City: Non-Parallel DNA Methylation Modifications across Pairs of Urban-Forest Great Tit Populations. Evol. Appl..

[65] Schulz B., Eckstein R.L., Durka W. (2014). Epigenetic Variation Reflects Dynamic Habitat Conditions in a Rare Floodplain Herb. Mol. Ecol..

[66] Shea N., Pen I., Uller T. (2011). Three Epigenetic Information Channels and Their Different Roles in Evolution. J. Evol. Biol..

[67] Herman J.J., Spencer H.G., Donohue K., Sultan S.E. (2014). How Stable “Should” Epigenetic Modifications Be? Insights from Adaptive Plasticity and Bet Hedging. Evolution.

[68] Herrera C.M., Medrano M., Bazaga P. (2014). Variation in DNA Methylation Transmissibility, Genetic Heterogeneity and Fecundity-Related Traits in Natural Populations of the Perennial Herb Helleborus Foetidus. Mol. Ecol..

[69] Stajic D., Jansen L.E.T. (2021). Empirical Evidence for Epigenetic Inheritance Driving Evolutionary Adaptation. Philos. Trans. R. Soc. B Biol. Sci..

[70] Zhou J., Zhu X., Cai Z. (2010). Tributyltin Toxicity in Abalone (Haliotis Diversicolor Supertexta) Assessed by Antioxidant Enzyme Activity, Metabolic Response, and Histopathology. J. Hazard. Mater..

[71] Ferraz da Silva I., Merlo E., Costa C.S., Graceli J.B., Rodrigues L.C.M. (2021). Tributyltin Exposure Is Associated with Recognition Memory Impairments, Alterations in Estrogen Receptor α Protein Levels, and Oxidative Stress in the Brain of Female Mice. Front. Toxicol..

[72] Shi Y., Chen C., Li M., Liu L., Dong K., Chen K., Qiu X. (2021). Oral Exposure to Tributyltin Induced Behavioral Abnormality and Oxidative Stress in the Eyes and Brains of Juvenile Japanese Medaka (Oryzias Latipes). Antioxidants.

[73] Primost M.A., Sabatini S.E., Di Salvatore P., Ríos De Molina M.C., Bigatti G. (2017). Oxidative Stress Indicators in Populations of the Gastropod Buccinanops Globulosus Affected by Imposex. J. Mar. Biol. Assoc. U. K..

[74] Hamilton P.B., Rolshausen G., Uren Webster T.M., Tyler C.R. (2016). Adaptive Capabilities and Fitness Consequences Associated with Pollution Exposure in Fish. Philos. Trans. R. Soc. B Biol. Sci..

[75] Madeira D., Mendonça V., Madeira C., Gaiteiro C., Vinagre C., Diniz M.S. (2019). Molecular Assessment of Wild Populations in the Marine Realm: Importance of Taxonomic, Seasonal and Habitat Patterns in Environmental Monitoring. Sci. Total Environ..

[76] Márquez F., González-José R., Bigatti G. (2011). Combined Methods to Detect Pollution Effects on Shell Shape and Structure in Neogastropods. Ecol. Indic..

[77] Primost M.A., Bigatti G., Márquez F. (2015). Shell Shape as Indicator of Pollution in Marine Gastropods Affected by Imposex. Mar. Freshw. Res..

