# Supplementary material for: Marine Pollutant Tributyltin Affects DNA Methylation and Fitness of Banded Murex (Hexaplex trunculus) Populations
Source: Toxics. 2023 Mar 17;11(3):276. doi: 10.3390/toxics11030276 (PMC10051066; doi:10.3390/toxics11030276)
Supplement: Supplementary file 1 [file toxics-11-00276-s001.zip › toxics-2220058-supplementary.pdf]

Supplementary Information for

Marine pollutant tributyltin affects DNA methylation and fitness of banded murex (*Hexaplex trunculus*) populations

Maja Šrut[1], Iva Sabolić[2], Anita Erdelez[3], Dorotea Grbin[2], Martina Furdek Turk[4], Robert Bakarić[2], Melita Peharda [3], Anamaria Štambuk[2]

[1]Institute of Zoology, Center for Molecular Biosciences, University of Innsbruck, Technikerstraße 25, 6020 Innsbruck, Austria

[2]Department of Biology, Faculty of Science, University of Zagreb, Rooseveltov trg 6, 10000 Zagreb, Croatia

[3]Institute of Oceanography and Fisheries, Šetalište I. Meštrovića 63, 21000 Split, Croatia

[4]Division for Marine and Environmental Research, Ruđer Bošković Institute, Bijenička 54, 10000 Zagreb, Croatia

Keywords: population epigenetics, fitness, imposex, pollution, evolution

Corresponding author:

Anamaria Štambuk

Department of Biology, Faculty of Science, University of Zagreb, Rooseveltov trg 6, 10000 Zagreb, Croatia

astambuk@biol.pmf.hr

Material and methods

Sampling and sample processing

Table S1. Total number of banded murex (*H. trunculus*) individuals collected in the Adriatic Sea and processed in each analysis.

Site	N total (♀ + ♂)	N morphometry (♀ + ♂)	N imposex (♀)	N OTCs (♀)	N biomarkers (♀ + ♂)	N MSAP (♀ + ♂)
Split-harbour	48 (33 + 15)	48	33	10	6	30
Vranjic	77 (29 + 48)	77	29	8	10	30
Trogir	60 (24 + 36)	60	24	10	10	30
Split-Špinut	59 (31 + 28)	59	31	10	10	30
Ston	86 (31 + 55)	86	31	10	8	30
Marina	88 (36 + 52)	88	36	10	10	30
Čiovo	54 (31 + 23)	54	31	9	10	30
**Total**	**472 (215 + 257)**	**472**	**215**	**67**	**64**	**210**

Biomarkers activity measurements

For protein extraction, the digestive gland tissue of 6–10 individuals per population was homogenized in an MM300 TissueLyser (Qiagen-Retsch) with steel balls in 1.2 mL of a 50 mM potassium phosphate buffer (pH 7.0) with 0.1 mM of EDTA. The homogenate was centrifuged at 10000 × g for 12 min at 4 °C. The supernatant was collected and used for biomarker activity measurements.

For enzyme assays, supernatants were diluted with an extraction buffer 1:5 (v/v). All measurements were performed with a SPECORD 40 spectrophotometer (Analytic Jena AG). Catalase (CAT) activity was assayed by measuring the decrease in absorbance at 240 nm ($\varepsilon = 36\ mM^{-1}cm^{-1}$) [31]. The reaction mixture consisted of a 50 mM potassium phosphate buffer (pH 7.0), 10 mM of H_2O_2, and 50 µL of the sample. CAT activity is expressed in units per mg of protein, where one unit is the amount of enzyme necessary for the hydrolysis of 1 mol of H_2O_2 per minute at 25 °C and pH 7.0. Glutathione reductase (GR) activity was determined via NADPH oxidation at 340 nm ($\varepsilon = 6.22\ mM^{-1}cm^{-1}$) [32]. The reaction mixture contained a 50 mM potassium phosphate buffer (pH 7.0), 0.1 mM of EDTA, 0.2 mM of NADPH, 0.5 mM of GSSG, and 100 µL of the sample. GR activity is expressed in units per mg of protein, where one unit is the amount of enzyme necessary for the oxidation of 1 nmol of NADPH per minute at 25 °C and pH 7.0. Glutathione S-transferase (GST) activity was assayed by measuring the decrease in absorbance at 340 nm ($\varepsilon = 9.6\ mM^{-1}cm^{-1}$), as previously described [33] with slight modifications. The reaction mixture consisted of a 50 mM

potassium phosphate buffer (pH 6.5), 0.1 mM of EDTA, 1 mM of reduced glutathione (GSH), 50 µL of the extract, and 500 µL of 1.5 mM 1-chloro-2.4-dinitrobenzene (CDNB) in ethanol (final concentration of 0.5 mM). GST activity is expressed as nmol of conjugated glutathione per minute per mg of protein. Acetylcholinesterase (AChE) activity was assayed by measuring the decrease in absorbance at 412 nm (ε = 0.07 $mM^{-1}cm^{-1}$), as previously described [34]. The reaction mixture consisted of a 100 mM potassium phosphate buffer (pH 7.4), 0.1 mM of EDTA, 8 mM of 5 5'-Dithiobis (2-nitrobenzoic acid) (DTNB), 8 mM of acetylcholine, and 50 µl of the sample. AChE activity is expressed as nmol of thiocholine released per minute per mg of protein. For carbonyl quantification, a dinitrophenylhydrazine (DNPH) reaction was used [35]. Briefly, 200 µL of supernatants diluted to 1:1 (v/v) with an extraction buffer were combined with 300 µL of 10 mM DNPH in 2 M of HCl. As a blind sample, 200 µL of an extract mixed with 2 M of HCl (300 µL) was used. After 1 h of incubation at room temperature (samples were mixed every 15 min), the proteins were precipitated with 500 µL of cold 10% (w/v) trichloroacetic acid, and the pellets were washed three times with 500 µL of ethanol:ethylacetate (1:1; v:v) and centrifuged at 12000 x g for 10 min at 4 °C to remove the excess reagent. The precipitated proteins were dissolved in 6 M of urea in a 20 mM potassium phosphate buffer (pH 2.4), and the absorption at 370 nm was measured. Protein recovery was estimated for each sample by measuring the absorption at 280 nm. Carbonyl contents were calculated using a molar absorption coefficient for aliphatic hydrazones (ε = 22 mM^{-1} cm^{-1}) and is expressed in µmol per mg of protein. The level of lipid peroxidation was indirectly determined as the formation of malondialdehyde (MDA) in a reaction with thiobarbituric acid (TBA), as previously described [36] with slight modifications. Homogenates (150 µL) were mixed with 450 µL of cold 10% trichloroacetic acid (TCA) to precipitate proteins. The precipitate was pelleted by centrifugation (10000 × g for 15 min at 4 °C), and 550 µL of the supernatant was reacted with an equal volume of 0.7% (w/v) TBA in 10% TCA. After heating at 90 °C for 15 min, the mixture was cooled in an ice bath. The absorbance of the supernatant was measured at 532 nm, and correction for unspecific turbidity was conducted by subtracting the absorbance at 600 nm. As a blind sample, 0.7% TBA in 10% TCA was used. The content of lipid peroxides was calculated using a molar absorption coefficient (ε = 155 mM^{-1} cm^{-1}) and is expressed as µmol per mg of protein. The total protein

content was determined with the Bradford method [37]. Values for each biomarker were normalized based on the protein content in each sample. A PCA of biomarker data was performed using R studio version 1.0.136. ANOVA and Tukey post-hoc tests were applied to PC1 and PC2 scores to define significance in population separation using the “aov” and “TukeyHSD” functions, respectively, in R studio ($p \leq 0.05$).

Results

Biomarkers

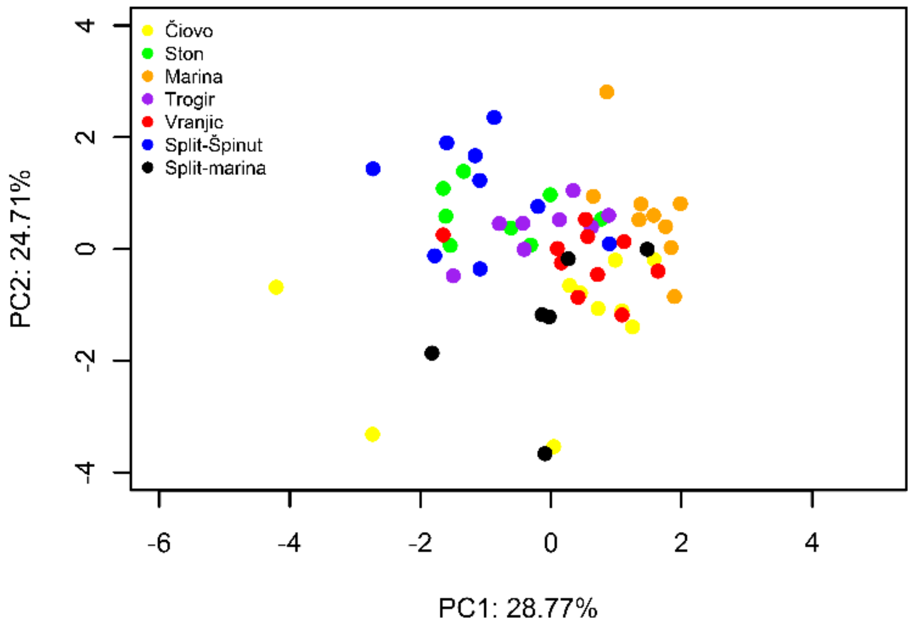

Figure S1. Principal component analysis (PCA) of *H. truncul
us* biomarker responses. In PC1, only the intermediately polluted site of Marina was significantly separated from the lowly and highly polluted sites; ANOVA and Tukey post-hoc test, $p \leq 0.05$; In PC2, there was no significant separation based on pollution intensity; ANOVA and Tukey post-hoc test, $p \leq 0.05$.

Morphometry

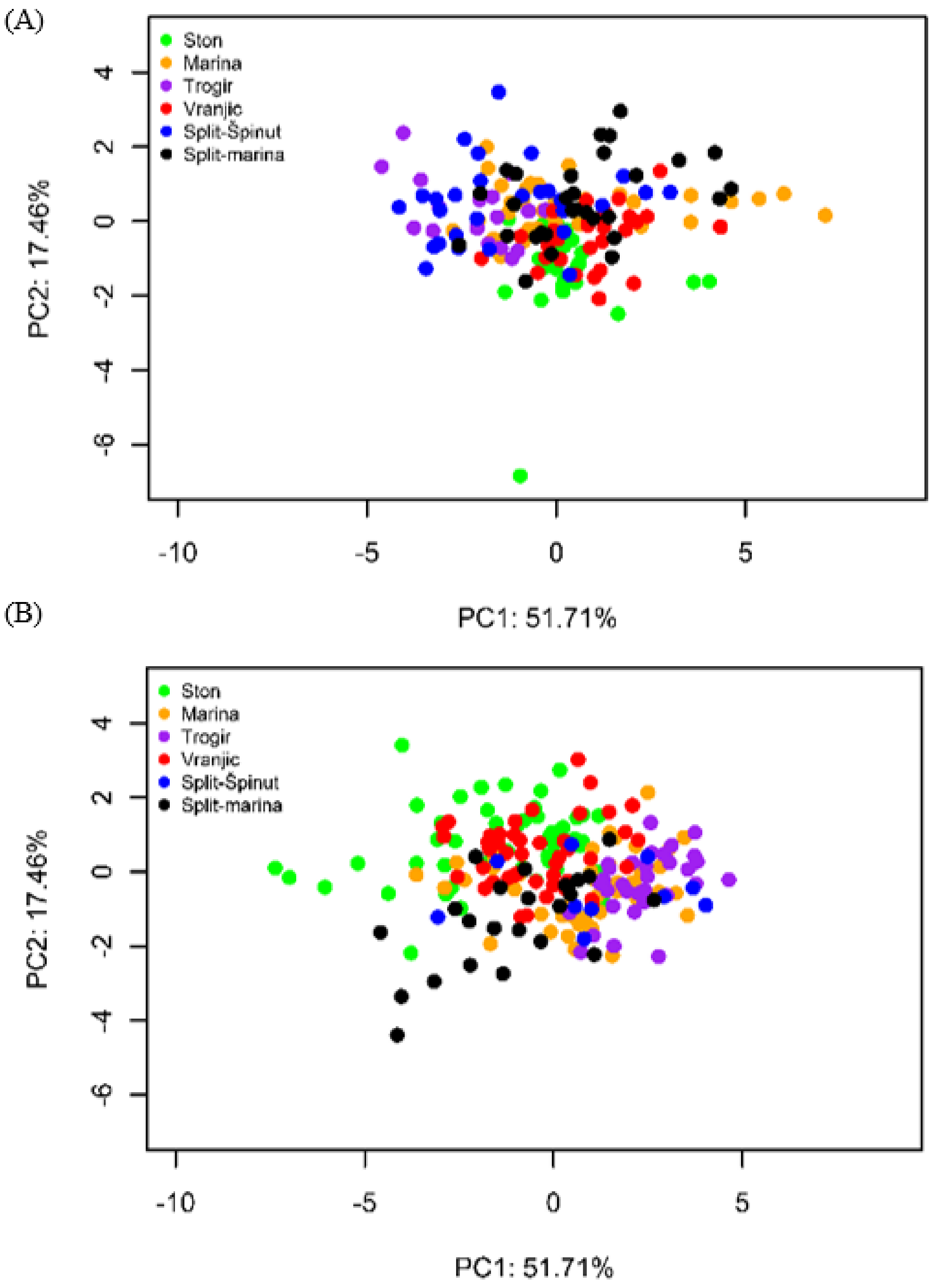

Figure S2. Principal component analysis (PCA) of *H. trunculus* morphometric measures. (A) Females: In PC1, there was a significant separation of snails originating from the highly polluted sites of Trogir and Split-Špinut from the other sites. In PC2, the lowly polluted site of Ston was significantly separated from the intermediately and highly polluted sites. (B) Males: In both PC1 and PC2, the lowly polluted site was significantly separated from the intermediately and highly polluted sites; ANOVA and Tukey post-hoc test, $p \leq 0.05$.

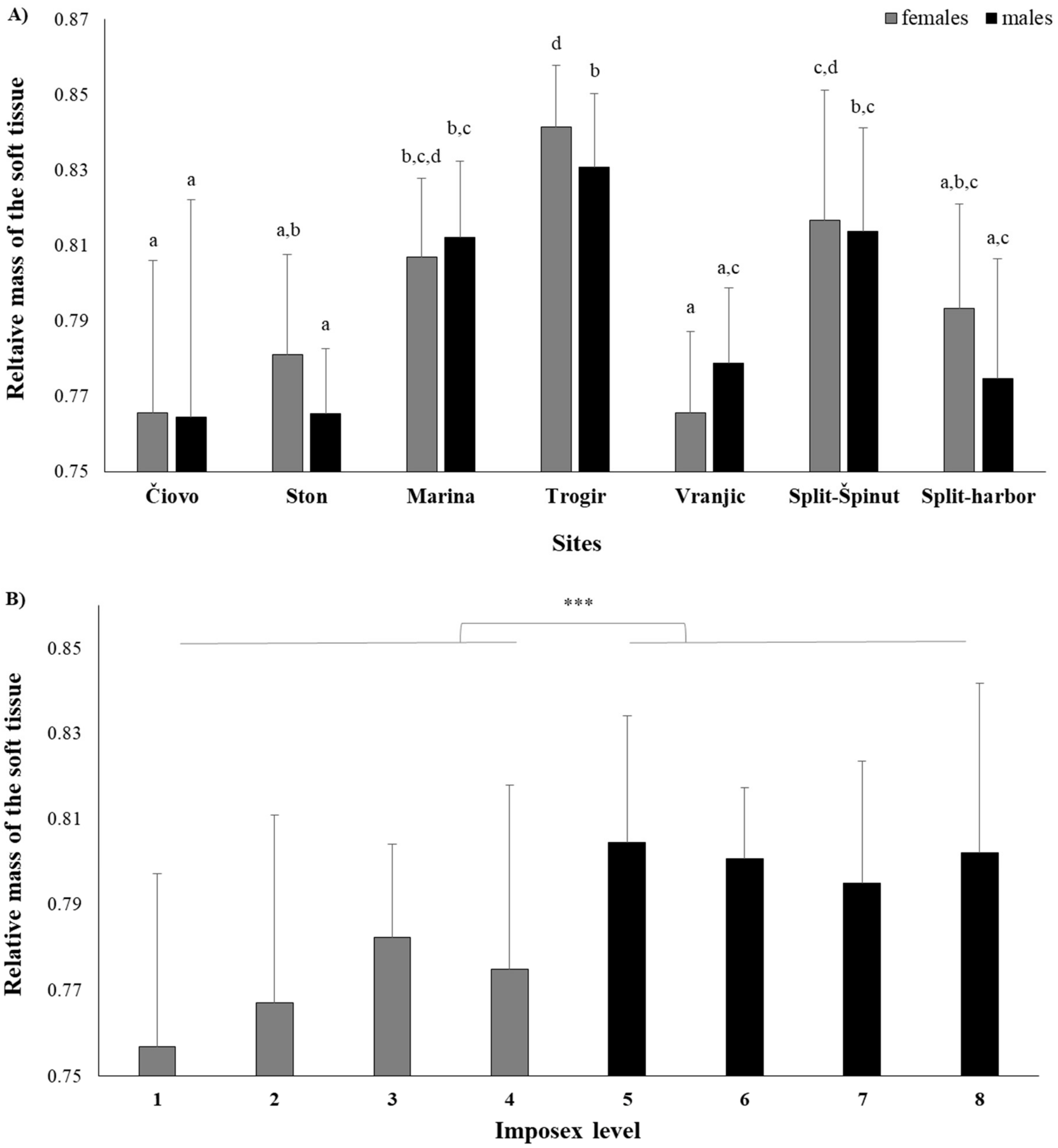

Figure S3. Relative mass of the soft tissue normalized on total animal length (log data); (A) per sampling site on females and males; (B) per imposex level across all populations. Letters relate to the significance between populations (for females and males separately) (ANOVA and Tukey post-hoc test, $p<0.05$), and stars denote the significance between low (1-4) and high (5-8) imposex levels (ANOVA and Tukey post-hoc test, $p<0.001$).

Epigenetic and genetic diversity

Table S2. Genetic diversity indices calculated for genetic (NML) and epigenetic (MSL) fragments. **A_N**—observed number of alleles; **A_E**—effective number of alleles; **h**—Nei's gene diversity; **I**—Shannon's information index.

		Genetic (NML)				Epigenetic (MSL)			
**Pollution level**	**Population**	**A_N**	**A_E**	**h**	**I**	**A_N**	**A_E**	**h**	**I**
**Low**	**Čiovo**	1.698	1.097	0.082	0.159	1.518	1.142	0.098	0.166
	**Ston**	1.395	1.052	0.044	0.085	1.501	1.130	0.088	0.151
**Intermediate**	**Marina**	1.581	1.074	0.062	0.123	1.558	1.165	0.109	0.182
**High**	**Trogir**	1.488	1.067	0.057	0.111	1.524	1.162	0.109	0.182
	**Vranjic**	1.419	1.057	0.048	0.095	1.597	1.160	0.108	0.184
	**Split-Špinut**	1.558	1.077	0.065	0.126	1.575	1.161	0.110	0.186
	**Split-harbour**	1.605	1.087	0.074	0.142	1.549	1.155	0.105	0.178

Table S3. Fragments putatively under selection (outliers) detected using Arlequin and Mcheza software: (A) epigenetic (MSL) fragments; (B) genetic (NML) fragments.

(A) Epigenetic (MSL) fragments

**Fragment ID**	**Arlequin**	**Mcheza**
**S1B_164**	+	-
**S1G_41**	+	-
**S1G_227**	+	-
**S2G_176**	+	+
**S3B_145**	+	+
**S3B_161**	-	+
**S3G_2**	+	-

(B) Genetic (NML) fragments

**Fragment ID**	**Arlequin**	**Mcheza**
**S1G_53**	+	+

References

31. Aebi, H. Catalase in Vitro. *Methods Enzymol.* **1984**, *105*, 121–126.
32. Ramos-Martinez, J.I.; Bartolomé, T.R.; Pernas, R.V. Purification and Properties of Glutathione Reductase from Hepatopancreas of Mytilus Edulis L. *Comp. Biochem. Physiol. Part B Comp. Biochem.* **1983**, *75*, 689–692, doi:10.1016/0305-0491(83)90117-7.
33. Habig, W.H.; Pabst, M.J.; Jakoby, W.B. Glutathione S-Transferases. The First Enzymatic Step in Mercapturic Acid Formation. *J. Biol. Chem.* **1974**, *249*, 7130–7139.
34. Ellman, G.L.; Courtney, K.D.; Andres, V.; Featherstone, R.M. A New and Rapid Colorimetric Determination of Acetylcholinesterase Activity. *Biochem. Pharmacol.* **1961**, *7*, 88–95, doi:10.1016/0006-2952(61)90145-9.
35. Levine, R.L.; Williams, J.A.; Stadtman, E.R.; Shacter, E. Carbonyl Assays for Determination of Oxidatively Modified Proteins. *Methods Enzymol.* **1994**, *233*, 346–357.
36. Buege, J.A.; Aust, S.D. Microsomal Lipid Peroxidation. *Methods Enzymol.* **1978**, *52*, 302–310.
37. Bradford, M.M. A Rapid and Sensitive Method for the Quantitation of Microgram Quantities of Protein Utilizing the Principle of Protein-Dye Binding. *Anal. Biochem.* **1976**, *72*, 248–254.
